# Structural Design and Verification of an Effective-Area Measurement Device Detection System

**DOI:** 10.3390/s23198215

**Published:** 2023-10-01

**Authors:** Xiangzi Chen, Ziping Yun, Ting You, Xiangqian Quan

**Affiliations:** 1 College of Marine Science and Technology, Hainan Tropical Ocean University, Sanya 572022, China; xzchen913@163.com (X.C.); y17530802036@163.com (Z.Y.); 18389264579@sina.cn (T.Y.); 2 Changchun Institute of Optics, Fine Mechanics and Physics, Chinese Academy of Sciences, Changchun 130033, China; 3 Yazhou Bay Innovation Institute, Hainan Tropical Ocean University, Sanya 572022, China; 4 Institute of Deep-Sea Science and Engineering, Chinese Academy of Sciences, Sanya 572000, China

**Keywords:** effective-area method, absolute solar irradiance radiometer, measurement device, design of detection system

## Abstract

The effective-area method is a new way to measure aperture area. It defines aperture area by directly using the beam-limiting effect of the aperture in radiometric measurement. Due to the special structure of the measurement device, it is necessary to find a suitable method to design the detection system. In this paper, the measurement system model is constructed in the TracePro program. The real circumstances of light propagation for the measurement beam are simulated, and the responses of the detector are given. It is proved that the relative change in the detector response is the lowest when the detector is at the position of 132°. And this is the best structure design of the detection system. The experimental results are designed to verify the feasibility of the structure design of the detection system.

## 1. Introduction

As the most important external energy source of the earth, the sun is the main driving force affecting the earth’s climate evolution and environmental change. It is necessary to monitor solar irradiance for a long time to provide accurate solar radiation data for the study of climate and environmental changes [1,2]. Changchun Institute of Optics, Fine Mechanics and Physics began to study absolute radiation measurement in the 1960s and developed a plane radiometer [3,4,5]. A cavity solar irradiation absolute radiometer (SIAR) was successfully developed in the 1990s [6,7]. A solar constant monitor (SCM), composed of SIARs, measured the solar irradiance data in the orbital module of the Shenzhou-3 spacecraft. Before the opening of the main cavity of the SIAR, an aperture requiring precision machining and area-measuring was placed [8].

According to the process of aperture area measurement, the existing methods are divided into the contact method and non-contact method [9,10]. The contact method for measuring the area is based on the usage of diameter-measuring machines. Diameter values at several points along the circular aperture are determined by translating a ball-ended stylus from one edge to the other. The edge of the measured aperture will be damaged in this method. The measurement accuracy is 0.15 × 10^−6^ m [8]. The non-contact method can be classified into the flux-compare method, optical-geometry method, and effective-area method [7]. The measurement accuracy of the flux-compare method [11,12], which requires a high-accuracy determination of the benchmark aperture area, is relatively low. This method is only used to measure small and difficult-to-measure apertures. The measurement accuracy of the optical-geometry method, which is mastered with the representative institutions of NPL and NIST [13,14,15], is high. But, it can only measure apertures of regulation shape (circular apertures). At the same time, the procedures for the measurement and the data processing are complex. The effective-area method is a way to measure the aperture area which uses the beam-limiting effect of the aperture. The measurement process is closer to the actual application process of the aperture, and the measurement accuracy is high [16,17]. This paper briefly introduces the measurement principle and measurement device of the effective-area method. According to the special structure of the measurement device, the optical model of the measurement system is established in Tracepro software. The real situation of measuring the light beam incident to the detection system is simulated. The response values of the detector at different positions relative to the entrance of the integrating sphere are analyzed. And a mathematical model is established to find the optimal position of the detector, so as to determine the optimal structure design of the detection system.

## 2. Measurement Principle and Measurement Device of the Effective-Area Method

### 2.1. Measurement Principle

The effective-area method is a non-contact method to measure the area of the aperture diaphragm, which defines the area of an aperture stop by taking advantage of the limiting effect of the aperture stop on the beam. In this method, a light source region with uniform illumination distribution is formed by superimposing multiple Gaussian beams, and the diaphragm area is defined by the limiting effect of the diaphragm on the beam in the radiation measurement. The area given by the measurement is called the effective area (not equivalent to the geometrical area of the stop). Only in the ideal circumstance do the geometric area and the effective area have the same value. The effective-area method solves the need to use the optical method to measure the main diaphragm area of the absolute solar radiometer. The measurement process of the main diaphragm area is consistent with the real use process of the main diaphragm area (the limiting effect of the size of the diaphragm area on the beam) and improves the measurement accuracy of the main diaphragm area. The measurement accuracy of solar irradiance measured using an absolute radiometer is indirectly improved. The aperture under test is placed in a uniformly distributed light source area with illumination E, and the plane of the aperture is perpendicular to the direction of beam propagation. A detector is used to receive the radiation flux P transmitted through the aperture. According to the basic relationship between radiation flux, irradiance, and area E = P/A, the area of the aperture A = P/E is calculated. We can combine and stack the exact same Gaussian-type laser beam to form a uniform illumination and uniform distribution of the light source region in the optical method. In fact, the known light source region with a uniform illuminance distribution can be replaced by fixing a laser beam and moving the measured aperture with constant steps ∆x and ∆y on the plane perpendicular to the propagation of the laser beam. By finding out the sum of all the light fluxes through the main aperture and finding the illumination of this equivalent uniform light source area, the aperture area can be obtained.

### 2.2. Measurement Devices

**Figure 1:**
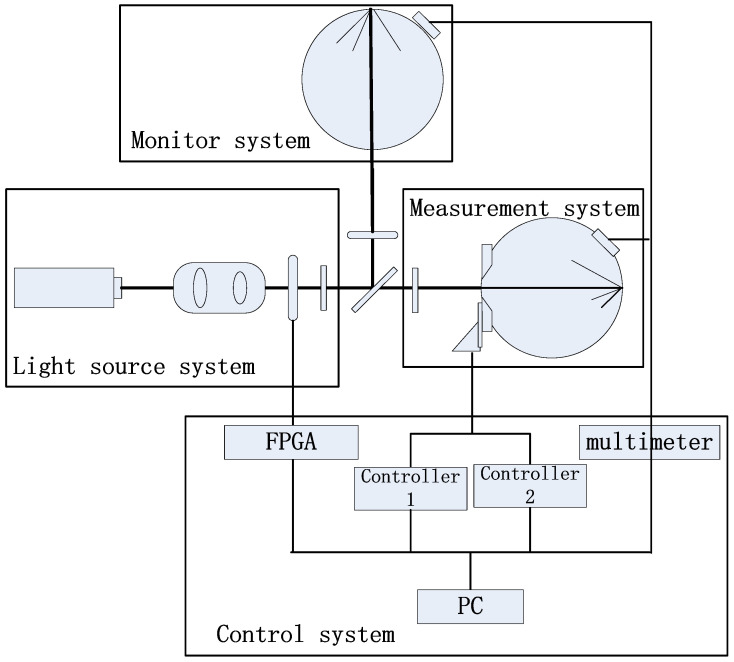
A diagram of the measurement device.

The effective-area method measurement device mainly includes four parts: a light source system, monitor system, measurement system, and control system. A schematic diagram of the measurement device is shown in Figure 1.

(1) The light source system is composed of a He-Ne laser, space filter, power stabilizer, iris blade, switch, beam separator, etc. The laser beam becomes a clean and stable Gaussian beam after passing through a spatial filter and a power stabilizer. The radiation power of the Gaussian beam is about 2 mw, and the long-term stability of the beam power is about 3.0 × 10^−5^ mw. The beam passes through the beam separator and becomes two light beams: one beam of light incident to the monitor system is called the reference-light, another incident to the measurement system is called the measurement-light. To ensure the stability of the light source output beam, an anti-stray diaphragm is added to eliminate the stray light which is brought by the surrounding environment and other factors.

(2) The monitor system is composed of an iris blade and an integrating sphere detector. It is used to monitor the stability of the Gaussian beam in the light source system. The monitoring system is applied to monitor the stability of the laser output Gaussian beam and provide monitoring data for the later data processing in the measurement process of measuring the area of the main diaphragm. The reference-light incident enters the integrating sphere detector and is converted into an electrical signal by a photodiode detector, which is measured with a multimeter device.
(3) The measurement system is composed of a two-dimensional translation stage, an integrating sphere detector, and an iris blade. It is used to measure the area of the aperture. The aperture is mounted on the translation stage, moving at fixed steps ∆x and ∆y in a plane perpendicular to the direction of Gaussian beam propagation. The measurement-light incident enters the integrating sphere detector through the entrance and is converted into an electrical signal by a photodiode detector, which is measured with a multimeter device. During the measurement process, a mirror is temporarily attached to the back of the measured main stop for adjusting the propagation direction of the measured beam to be perpendicular to the plane where the measured main stop is located. After the light path is adjusted, the plane mirror is removed.
(4) The control system is used to control the closing of the switch, control the motion of the two-dimensional precision electronic translation platform, and control the acquisition of radiation flux data from the detector in the measurement system and monitoring system. The control system makes each sub-system cooperate, realizes the measurement and recoding of the data, and finally completes the whole experimental measurement process. The program of the control system is written with the software of LabVIEW. The whole experiment adopts a fully automatic operation mode without human intervention.

## 3. Structural Model Design of the Detection System

**Figure 2:**
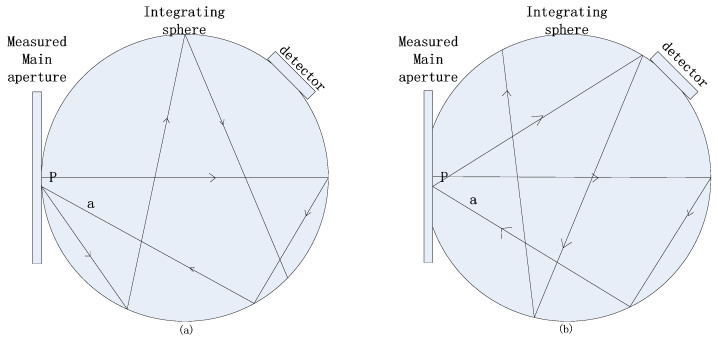
The diagram of the measurement-light spreading. (**a**) The back of the measured aperture does not serve as part of the sphere wall, the measurement-light occurs as a diffuse reflection at point P. (**b**) The back of the measured aperture serves as part of sphere wall, the measurement-light occurs as a mirror reflection at point P.

In the measurement system, the measurement-light passes through the measured aperture and enters into the integrating sphere detector. The aperture carried on the translation platform moves at a fixed stride distance. The position and propagation direction of the measurement-light remain unchanged. When the measurement-light hits the edge of the aperture, scattering will occur. All the edge scattered lights need to be collected and detected by the detection system. Due to the large divergence angle of the edge scattered light, the entrance of the integrating sphere needs to be fully large to ensure that the measured aperture rim is all contained inside it. According to the design principle of the integrating sphere, the ratio of the integrating sphere opening area to the total surface area is less than 5%. During the measurement process, the polished aperture back will act as part of the inner wall of the integrating sphere, as shown in Figure 2. The arrow represents the propagation direction of light. The mirror reflection will occur when the measurement-light incident reaches the back of the aperture. But diffuse reflection will occur when the measurement-light incident reaches the wall of the integrating sphere differently. This difference affects the uniformity of the response of the integrating sphere. According to the previous experience of the integrating sphere design, the structure’s position between the integrating sphere entrance and the detector is vertical. For the special structure of the measurement system, it is necessary to design the position relationship between the entrance of the integrating sphere and the detector, minimizing the uniformity of the response which is brought by the back of the aperture acting as part of the integrating sphere wall. In the experimental design, the inner wall diameter of the integrating sphere is 100 mm, the entrance diameter is 25 mm, the inner wall attribute of the integrating sphere is the Lambert diffuse reflector, and the reflectivity is 97%.

### 3.1. Structure Model of the Measurement System

**Figure 3:**
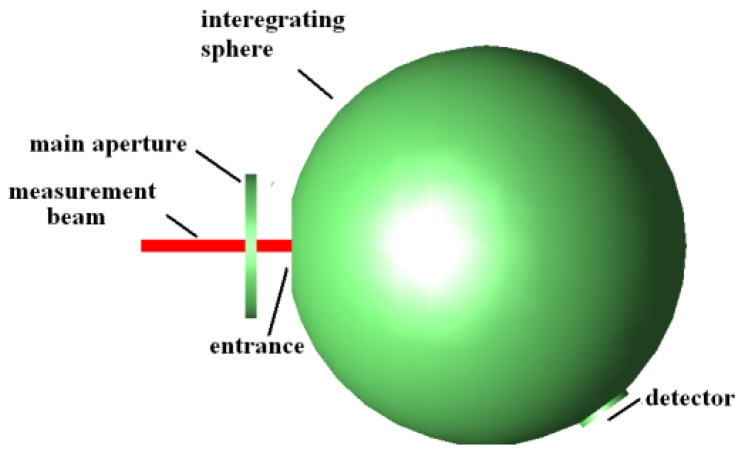
A diagram of the measurement system structure model.

The measurement system structure model was built in the TracePro program. The measurement-light, the measured aperture, and the integrating sphere are on the same axis. The measurement-light passes through the measured aperture and hits the inside of the integrating sphere. The back of the measured aperture acts as part of the inner wall of the integrating sphere; a structural model is shown in Figure 3. The distance *M* between the measured aperture and the integrating sphere is set to *M* = 0, 0.5, 0.75, 1, 1.25, or 1.5, respectively. And six different measurement system structure models are established in the TracePro program.

### 3.2. Structure Model of the Detection System

**Figure 4:**
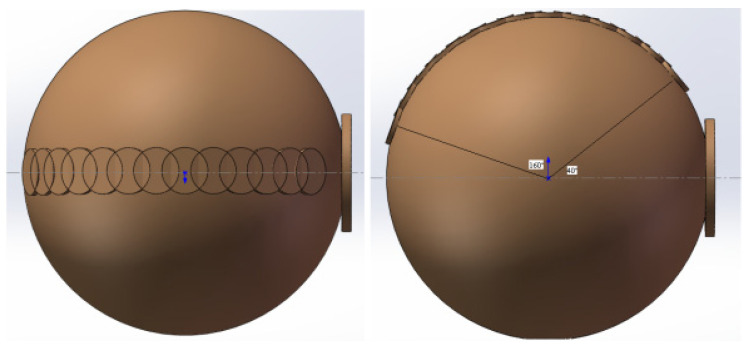
The structure models of the detector systems with the detector in different positions.

The detection system structure models were established on the basis of six different measurement system structure models. For each measurement system, the detector was designed in a different position relative to the integrating sphere entrance. The positions of the detectors are distributed on the plane perpendicular to the integrating sphere entrance. On the entrance side of the integrating sphere, thirteen different positions were designed. The angle between the vertical line of the detector and the vertical line of the integrating sphere entrance ranges from the 40° position to 160° position, where each position is 10° apart. Each detector position corresponds to a detection system model, as shown in Figure 4.

## 4. Analysis of Simulation Results of Detection System

In the TracePro program, the measurement-light is set to a Gaussian distribution and the total luminous flux is 2.7 w. Six measurement system structure models and thirteen detection system structure models are combined into seventy-eight different integrating sphere detection simulation models. The response value of the detector to the measurement-light is $P_{M}^{i}$, where the *M* is 0 mm, 0.5 mm, 0.75 mm, 1 mm, 1.25 mm, or 1.5 mm and the *i* is 1, 2, 3, 4, 5, 6, 7, 8, 9, 10, 11, 12, or 13. Each *M* value corresponds to a measurement system model, and each *i* value corresponds to a detection system model. The measurement system with zero distance between the measured aperture and the integrating sphere entrance (that is, *M* = 0) is assumed to be the reference measurement system, and the response value of the detector $P_{0}^{i}$ is referred to as the reference standard. The relative change in the detector response value of each detection system is determined with the formula

(1) $$r^{i}{}_{M}=(P_{0}^{i}-P_{M}^{i})/P_{0}^{i}$$

where *M* is 0.5 mm, 0.75 mm, 1 mm, or 1.25 mm and *i* is 1, 2, 3, 4, 5, 6, 7, 8, 9, 10, 11, 12, or 13. For each measurement system, the relative change in the detector response value is summed by the formula

(2) $$R_{M}=\sum_{i=1}^{13}r^{i}{}_{M}$$

where *M* is 0.5 mm, 0.75 mm, 1 mm, or 1.25 mm. Thus, the detector position A is found when the sum of the detector response values is the minimum value $r_{M(i)}^{\min}$. Ten different detector positions j are redesigned to the left and right of position A. Each position is 2 degrees apart. Sixty integrating sphere detection simulation models are re-established. And the position B of the detector is found when the sum of the detector response values is the minimum using the same method. Then, the optimal structural design of the detection system is determined.

**Figure 5:**
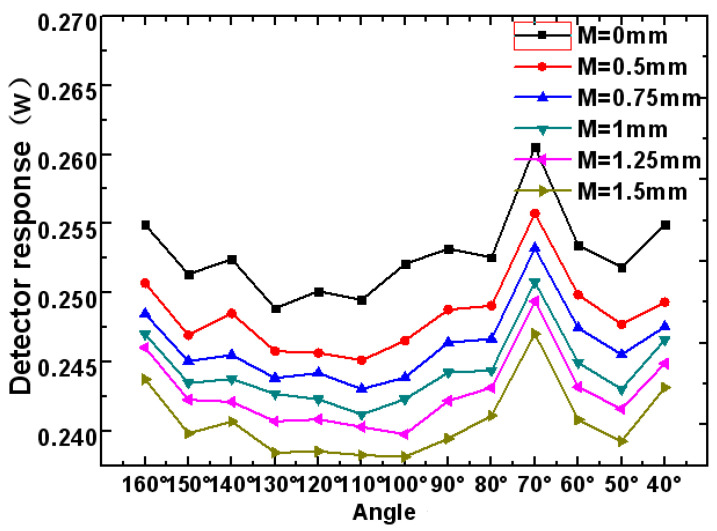
The curves of detector responses with different detection systems.

**Figure 6:**
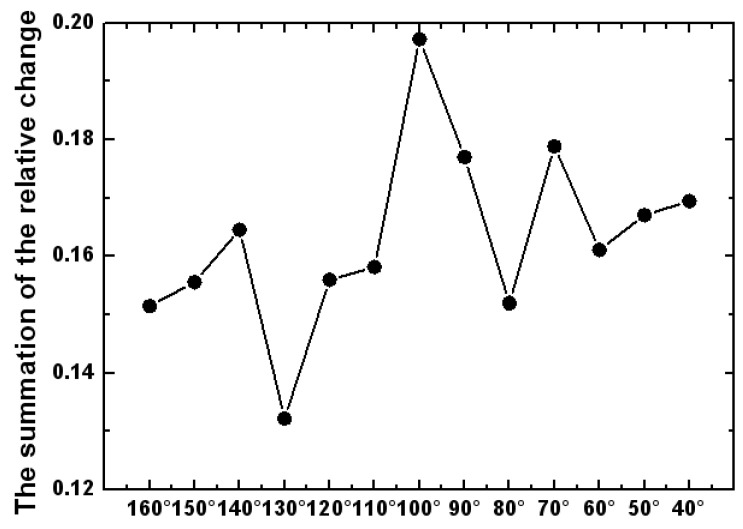
The curve of relative change in the detector response for all the different detection systems.

**Figure 7:**
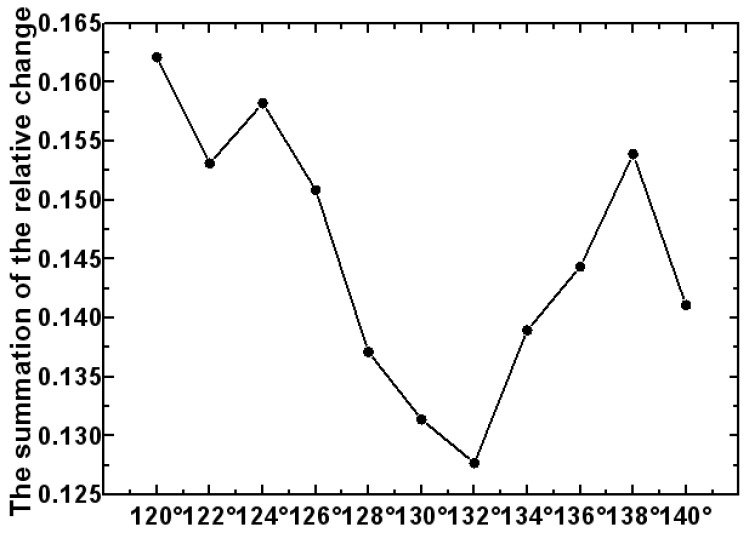
The relative change in the detector response with different positions.

(1) Light tracing in TracePro software was used to record the response value of the detector to the measurement-light $P_{M}^{i}$ under different integrating sphere detection simulation models. The detector response data curve of each different detection system is shown in Figure 5.
(2) When the distance between the measured aperture and the integrating sphere entrance is 0 mm (that is, *M* = 0 mm), the measurement system is the reference measurement system. And the corresponding detector response values $P_{0}^{i}$ of the different detection systems are the reference standards. According to Formula (1), the relative changes in the detector response values of all the different detection systems are found.
(3) According to Formula (2), the sum of the relative change in the response values under each detection system can be obtained. As shown in Figure 6, the position A of the detector with the minimum sum value $r_{M(i)}^{\min}$ is found, where the value is 130°.
(4) Due to the values remaining continuous and not changing drastically, we first find the minimum range in Figure 6. Then, we narrow down the scope in Figure 7. Ten different detector positions j are redesigned to the left and right of position A. Each position is 2 degrees apart. Sixty integrating sphere detection simulation models are re-established. We repeat the above steps of (1), (2) and (3), and the responses of all the detection systems to the measurement-light $P_{M}^{j}$ can be obtained. The position B of the detector with the minimum sum value $r_{M(j)}^{\min}$ of the detector response is found, as shown in Figure 7, and the detector is at the position of 132°. Then, the optimal structure design of the detection system is determined.

## 5. Experimental Test

Based on the above design of the detection system, a customized integrating sphere was designed and an effective-area method measurement device was built. The integrating sphere is customized with a diameter of 100 mm. And the inner wall is sprayed with a high-quality F4 diffuse reflective coating with a reflection of 97%. The detector is an S1227 series silicon detector (S1227-1010BQ) produced by the company HAMAMATSU. And the detector size is 15 × 16.5 mm^2^ with an effective detection of 10 × 10 mm^2^. The maximum reverse voltage is 5 V and the operating temperature is −20 °C~60 °C. The laser source uses a THORLABS HNL210L He-ne laser with a Gaussian beam wavelength of 632.8 nm and a beam waist diameter of 0.7 mm. The power stabilizer belongs to the Brockton Electro Optics LS-PRO series. The beam separator uses a spectroscope, which is a semi-transparent and semi-reflective mirror, for the wavelength of 632.8 nm. The aperture of the SIAR, with a nominal diameter of 5 mm, was measured. The measurement results are shown in Table 1. The repeatability of the aperture area measurement data reached 7 × 10^−5^. The area of the aperture is calibrated using a universal tool microscope (model19JA840087), which measures the diameter values of the aperture in different directions. According to the relationship between the area and the diameter, the aperture area is calculated indirectly. And the calibration area value of the aperture was 21.127 mm^2^. The measurement setup was designed according to the simulation where the integrating sphere was customized. Due to the processing and adjustment accuracy, the experiment could not achieve the coincident result in the simulation. Even so, the experiment results also certified our design. Comparing the aperture area measurement results of the two methods, the relative deviation is 6.6 × 10^−5^, which proves that the measurement device is stable and reliable, and the structural design of the detection system is reasonable.

## 6. Discussion

The effective-area method is a non-contact method to measure the area of the aperture diaphragm which defines the area of the aperture stop by taking advantage of the limiting effect of the aperture stop on the beam, which is closer to the actual application process of the aperture, and the measurement accuracy is higher. One limitation of the measurement system is that the back of the main stop being measured will act as part of the inner wall of the integrating sphere (the inner wall of the opening) because of the opening of the integrating sphere. The main stop being measured is made of carbon steel material and its back is polished like a flat mirror (i.e., the flat mirror will act as part of the inner wall of the integrating sphere). Consequently, the light incident on the opening of the integrating sphere detector through diffuse reflection will be a specular reflection on the back surface of the main aperture when the measuring beam incident hits the inside of the integrating sphere detector, which is completely different from the diffuse reflection of the light incident hitting the inner surface of the integrating sphere. The phenomenon that the back of the measured main stop acts as the inner wall of the integrating sphere will seriously affect the uniformity of the response of the integrating sphere detector, and the non-uniformity of the response of the integrating sphere detector will affect the measurement results of the measured area of the main stop. Therefore, it is necessary to correct the measurement results of the effective-area method for measuring the main stop area of the absolute solar radiometer. The main problem to be solved in the future is to correct the problem that the back of the main stop acts as the inner wall of the integral sphere, which affects the measurement results. The main future research contents to be conducted include the design of a revised model and the research of a revised method. Firstly, the effect of the back of the main stop (polished) acting as the inner wall of the detector on the measurement results of the main stop area will be analyzed. Secondly, the structural model of the measurement system will be established in the optical software TracePro and the different modified structural models should be designed based on the measurement system model. The optimal corrected model should be obtained with simulations and the correction method and correction coefficient will be derived. Finally, based on the correction coefficient, the measured data of the effective-area method will be modified, and the corrected value of the measured area of the main diaphragm will be derived.

## 7. Conclusions

The effective-area method solves the need to measure the area of the main diaphragm using the optical method. We studied the measuring principle of the effective-area method and present the concrete measuring idea. With the overall measurement scheme design, the measuring device of the effective-area method was built, the measuring process of the effective-area method was designed, and the measuring result of the main diaphragm area was derived. The uncertainty of the average measurement result, which was caused by the measurement repeatability, meets the measuring accuracy requirement of the main diaphragm area in the absolute solar radiometer. The optical model of the detection system was established in the TracePro software to simulate a real situation of a measurement-light incident in the detection system. The response values of the detector to the measurement-light were analyzed in detail when the detector was in different positions relative to the entrance of the integrating sphere. The reference measurement system is specified when the distance between the measured aperture and the entrance of the integrating sphere is zero. Several measurement systems with different distances between the measured aperture and the entrance of the integrating sphere were simulated, and mathematical models were established. When the detector is at the position of 132°, the sum of the changes in the detector response values is the smallest. This is the optimal structural design of the detection system. The experimental results verify the feasibility of the structural design of the detection system. The effective-area method is a non-contact method to measure the area of the aperture diaphragm. The effective surface method defines the area of the aperture stop by taking advantage of the limiting effect of the aperture stop on the beam. In this method, a light source region with uniform illumination distribution is formed by superimposing multiple Gaussian beams, and the diaphragm area is defined by the limiting effect of the diaphragm on the beam in the radiation measurement. The area given by the measurement is called the effective area (not equivalent to the geometrical area of the stop). Only in very ideal cases do the geometric area and the effective area have the same value. The effective-area method solves the need to use the optical method to measure the main diaphragm area of the absolute solar radiometer. The measurement process of the main diaphragm area is consistent with the real use process of the main diaphragm area (the limiting effect of the size of the diaphragm area on the beam) and improves the measurement accuracy of the main diaphragm area. The measurement accuracy of solar irradiance measured using an absolute radiometer is improved indirectly.

## Figures and Tables

**Table 1:** The aperture area measurement results of this method.

No.	Measured Value (mm^2^)	No.	Measured Value (mm^2^)
1	21.1280	5	21.1268
2	21.1321	6	21.1262
3	21.1331	7	21.1221
4	21.1307		
RMS (mm^2^)	21.1284		
Standard deviation (mm^2^)	0.0038		

## Data Availability

The data presented in this study are available from the corresponding author upon request.
